# Supplementary material for: Development of a computer-aided design software for the quantitative evaluation of aesthetic damage
Source: PLoS One. 2019 Dec 18;14(12):e0226322. doi: 10.1371/journal.pone.0226322 (PMC6919621; doi:10.1371/journal.pone.0226322)
Supplement: S1 Code site — 1. (ZIP) [file pone.0226322.s001.zip › [EESC_jr][Projeto]Relatorio_07_21/reports/templates_zip/gentelella-master.zip!/gentelella-master/vendors/DateJS/reports/lcov-report/core/core-prototypes.js.html]

Code coverage report for core/core-prototypes.js


# Code coverage report for core/core-prototypes.js

## Statements: 95.1% (291 / 306)      Branches: 83.33% (195 / 234)      Functions: 100% (50 / 50)      Lines: 96.28% (285 / 296)      Ignored: none

All files » core/ » core-prototypes.js

```
|  |  |  |
| --- | --- | --- |
| 1 2 3 4 5 6 7 8 9 10 11 12 13 14 15 16 17 18 19 20 21 22 23 24 25 26 27 28 29 30 31 32 33 34 35 36 37 38 39 40 41 42 43 44 45 46 47 48 49 50 51 52 53 54 55 56 57 58 59 60 61 62 63 64 65 66 67 68 69 70 71 72 73 74 75 76 77 78 79 80 81 82 83 84 85 86 87 88 89 90 91 92 93 94 95 96 97 98 99 100 101 102 103 104 105 106 107 108 109 110 111 112 113 114 115 116 117 118 119 120 121 122 123 124 125 126 127 128 129 130 131 132 133 134 135 136 137 138 139 140 141 142 143 144 145 146 147 148 149 150 151 152 153 154 155 156 157 158 159 160 161 162 163 164 165 166 167 168 169 170 171 172 173 174 175 176 177 178 179 180 181 182 183 184 185 186 187 188 189 190 191 192 193 194 195 196 197 198 199 200 201 202 203 204 205 206 207 208 209 210 211 212 213 214 215 216 217 218 219 220 221 222 223 224 225 226 227 228 229 230 231 232 233 234 235 236 237 238 239 240 241 242 243 244 245 246 247 248 249 250 251 252 253 254 255 256 257 258 259 260 261 262 263 264 265 266 267 268 269 270 271 272 273 274 275 276 277 278 279 280 281 282 283 284 285 286 287 288 289 290 291 292 293 294 295 296 297 298 299 300 301 302 303 304 305 306 307 308 309 310 311 312 313 314 315 316 317 318 319 320 321 322 323 324 325 326 327 328 329 330 331 332 333 334 335 336 337 338 339 340 341 342 343 344 345 346 347 348 349 350 351 352 353 354 355 356 357 358 359 360 361 362 363 364 365 366 367 368 369 370 371 372 373 374 375 376 377 378 379 380 381 382 383 384 385 386 387 388 389 390 391 392 393 394 395 396 397 398 399 400 401 402 403 404 405 406 407 408 409 410 411 412 413 414 415 416 417 418 419 420 421 422 423 424 425 426 427 428 429 430 431 432 433 434 435 436 437 438 439 440 441 442 443 444 445 446 447 448 449 450 451 452 453 454 455 456 457 458 459 460 461 462 463 464 465 466 467 468 469 470 471 472 473 474 475 476 477 478 479 480 481 482 483 484 485 486 487 488 489 490 491 492 493 494 495 496 497 498 499 500 501 502 503 504 505 506 507 508 509 510 511 512 513 514 515 516 517 518 519 520 521 522 523 524 525 526 527 528 529 530 531 532 533 534 535 536 537 538 539 540 541 542 543 544 545 546 547 548 549 550 551 552 553 554 555 556 557 558 559 560 561 562 563 564 565 566 567 568 569 570 571 572 573 574 575 576 577 578 579 580 581 582 583 584 585 586 587 588 589 590 591 592 593 594 595 596 597 598 599 600 601 602 603 604 605 606 607 608 609 610 611 612 613 614 615 616 617 618 619 620 621 622 623 624 625 626 627 628 629 630 631 632 633 634 635 636 637 638 639 640 641 642 643 644 645 646 647 648 649 650 651 652 653 654 655 656 657 658 659 660 661 662 663 664 665 666 667 668 669 670 671 672 673 674 675 676 677 678 679 680 681 682 683 684 685 686 687 688 689 690 691 692 693 694 695 696 697 698 699 700 701 702 703 704 705 706 707 708 709 710 711 712 713 714 715 716 717 718 719 720 721 722 723 724 725 726 727 728 729 730 731 732 733 734 735 736 737 738 739 740 741 742 743 744 745 746 747 748 749 750 751 752 753 754 755 756 757 758 759 760 761 762 763 764 765 766 767 768 769 | 1 1     4331 2871   4331     1 151 151 335 119 119 119   216     151 565 565   565 330       150             1 2100 2100 2100 2100 2100             1 3 3 3 3 3 3           1 25               1 408               1 398                 1 1               1 2               1 2                           1 2               1 155 155 155               1 70 70               1 48 46               1 37 37               1 1851 1841 1841               1 14 12 12 12 4 2 2 2       12 5 11 11 11 5     5 7 2     7               1 55 54                 1 413 396 396 396 396 396     1 1       1               1 190 174                           1 508 83 83     425   425             425 1   425 31   425 31   425 31   425 1   425 109   425 109   425 119   425                 1   86 86 2 2   84       86       86   86     86   86 86       86               1 2               1 3 1     1     2       1 1 1     1 14     1 1                 1 15 5 2 2 2   3 2 2 1     1   4   10 10 8   2 2 2 1   2   8       1 2 1183 1183                 1             1         1 1 1           1 1             1 1     1 5 5     1 1             1 3             1 7             1 3 3 2 2   1 1                 1 19                             1 151 150 150 330 330 330 330 226   330 330 330 100 230 69   330 177 153 34         150 119     150             1 11             1 10                                                                                                                       1 7       1     1     1   4     1 1470 1470   1450   1   1   1     2 2   1   1   1   1 1   1   10     1 1498 4446 3   4443   2   4   5   1   11   1   7   1   1460   2         3   3   1424   39   5   3   1418   37   1   5   6   2   1   1   1                                       1       2958 1470 1470 1460     1498 1498 | ``` (function () { 	var $D = Date, 		$P = $D.prototype, 		p = function (s, l) { 			if (!l) { 				l = 2; 			} 			return ("000" + s).slice(l * -1); 		};   	var validateConfigObject = function (obj) { 		var result = {}, self = this, prop, testFunc; 		testFunc = function (prop, func, value) { 			if (prop === "day") { 				var month = (obj.month !== undefined) ? obj.month : self.getMonth(); 				var year = (obj.year !== undefined) ? obj.year : self.getFullYear(); 				return $D[func](value, year, month); 			} else { 				return $D[func](value); 			} 		}; 		for (prop in obj) { 			Eif (hasOwnProperty.call(obj, prop)) { 				var func = "validate" + prop.charAt(0).toUpperCase() + prop.slice(1);   				if ($D[func] && obj[prop] !== null && testFunc(prop, func, obj[prop])) { 					result[prop] = obj[prop]; 				} 			} 		} 		return result; 	}; 	/** 	 * Resets the time of this Date object to 12:00 AM (00:00), which is the start of the day. 	 * @param {Boolean}  .clone() this date instance before clearing Time 	 * @return {Date}    this 	 */ 	$P.clearTime = function () { 		this.setHours(0); 		this.setMinutes(0); 		this.setSeconds(0); 		this.setMilliseconds(0); 		return this; 	};   	/** 	 * Resets the time of this Date object to the current time ('now'). 	 * @return {Date}    this 	 */ 	$P.setTimeToNow = function () { 		var n = new Date(); 		this.setHours(n.getHours()); 		this.setMinutes(n.getMinutes()); 		this.setSeconds(n.getSeconds()); 		this.setMilliseconds(n.getMilliseconds()); 		return this; 	}; 	/** 	 * Returns a new Date object that is an exact date and time copy of the original instance. 	 * @return {Date}    A new Date instance 	 */ 	$P.clone = function () { 		return new Date(this.getTime()); 	};   	/** 	 * Compares this instance to a Date object and returns an number indication of their relative values.   	 * @param {Date}     Date object to compare [Required] 	 * @return {Number}  -1 = this is lessthan date. 0 = values are equal. 1 = this is greaterthan date. 	 */ 	$P.compareTo = function (date) { 		return Date.compare(this, date); 	};   	/** 	 * Compares this instance to another Date object and returns true if they are equal.   	 * @param {Date}     Date object to compare. If no date to compare, new Date() [now] is used. 	 * @return {Boolean} true if dates are equal. false if they are not equal. 	 */ 	$P.equals = function (date) { 		return Date.equals(this, (date !== undefined ? date : new Date())); 	};   	/** 	 * Determines if this instance is between a range of two dates or equal to either the start or end dates. 	 * @param {Date}     Start of range [Required] 	 * @param {Date}     End of range [Required] 	 * @return {Boolean} true is this is between or equal to the start and end dates, else false 	 */ 	$P.between = function (start, end) { 		return this.getTime() >= start.getTime() && this.getTime() <= end.getTime(); 	};   	/** 	 * Determines if this date occurs after the date to compare to. 	 * @param {Date}     Date object to compare. If no date to compare, new Date() ("now") is used. 	 * @return {Boolean} true if this date instance is greater than the date to compare to (or "now"), otherwise false. 	 */ 	$P.isAfter = function (date) { 		return this.compareTo(date \|\| new Date()) === 1; 	};   	/** 	 * Determines if this date occurs before the date to compare to. 	 * @param {Date}     Date object to compare. If no date to compare, new Date() ("now") is used. 	 * @return {Boolean} true if this date instance is less than the date to compare to (or "now"). 	 */ 	$P.isBefore = function (date) { 		return (this.compareTo(date \|\| new Date()) === -1); 	};   	/** 	 * Determines if the current Date instance occurs today. 	 * @return {Boolean} true if this date instance is 'today', otherwise false. 	 */ 	 	/** 	 * Determines if the current Date instance occurs on the same Date as the supplied 'date'.  	 * If no 'date' to compare to is provided, the current Date instance is compared to 'today'.  	 * @param {date}     Date object to compare. If no date to compare, the current Date ("now") is used. 	 * @return {Boolean} true if this Date instance occurs on the same Day as the supplied 'date'. 	 */ 	$P.isToday = $P.isSameDay = function (date) { 		return this.clone().clearTime().equals((date \|\| new Date()).clone().clearTime()); 	}; 	 	/** 	 * Adds the specified number of milliseconds to this instance.  	 * @param {Number}   The number of milliseconds to add. The number can be positive or negative [Required] 	 * @return {Date}    this 	 */ 	$P.addMilliseconds = function (value) { 		Iif (!value) { return this; } 		this.setTime(this.getTime() + value * 1); 		return this; 	};   	/** 	 * Adds the specified number of seconds to this instance.  	 * @param {Number}   The number of seconds to add. The number can be positive or negative [Required] 	 * @return {Date}    this 	 */ 	$P.addSeconds = function (value) { 		Iif (!value) { return this; } 		return this.addMilliseconds(value * 1000); 	};   	/** 	 * Adds the specified number of seconds to this instance.  	 * @param {Number}   The number of seconds to add. The number can be positive or negative [Required] 	 * @return {Date}    this 	 */ 	$P.addMinutes = function (value) { 		if (!value) { return this; } 		return this.addMilliseconds(value * 60000); // 60*1000 	};   	/** 	 * Adds the specified number of hours to this instance.  	 * @param {Number}   The number of hours to add. The number can be positive or negative [Required] 	 * @return {Date}    this 	 */ 	$P.addHours = function (value) { 		Iif (!value) { return this; } 		return this.addMilliseconds(value * 3600000); // 60*60*1000 	};   	/** 	 * Adds the specified number of days to this instance.  	 * @param {Number}   The number of days to add. The number can be positive or negative [Required] 	 * @return {Date}    this 	 */ 	$P.addDays = function (value) { 		if (!value) { return this; } 		this.setDate(this.getDate() + value * 1); 		return this; 	};   	/** 	 * Adds the specified number of weekdays (ie - not sat or sun) to this instance.  	 * @param {Number}   The number of days to add. The number can be positive or negative [Required] 	 * @return {Date}    this 	 */ 	$P.addWeekdays = function (value) { 		if (!value) { return this; } 		var day = this.getDay(); 		var weeks = (Math.ceil(Math.abs(value)/7)); 		if (day === 0 \|\| day === 6) { 			if (value > 0) { 				this.next().monday(); 				this.addDays(-1); 				day = this.getDay(); 			} 		}   		if (value < 0) { 			while (value < 0) { 				this.addDays(-1); 				day = this.getDay(); 				if (day !== 0 && day !== 6) { 					value++; 				} 			} 			return this; 		} else if (value > 5 \|\| (6-day) <= value) { 			value = value + (weeks * 2); 		}   		return this.addDays(value); 	};   	/** 	 * Adds the specified number of weeks to this instance.  	 * @param {Number}   The number of weeks to add. The number can be positive or negative [Required] 	 * @return {Date}    this 	 */ 	$P.addWeeks = function (value) { 		if (!value) { return this; } 		return this.addDays(value * 7); 	};     	/** 	 * Adds the specified number of months to this instance.  	 * @param {Number}   The number of months to add. The number can be positive or negative [Required] 	 * @return {Date}    this 	 */ 	$P.addMonths = function (value) { 		if (!value) { return this; } 		var n = this.getDate(); 		this.setDate(1); 		this.setMonth(this.getMonth() + value * 1); 		this.setDate(Math.min(n, $D.getDaysInMonth(this.getFullYear(), this.getMonth()))); 		return this; 	};   	$P.addQuarters = function (value) { 		Iif (!value) { return this; } 		// note this will take you to the same point in the quarter as you are now. 		// i.e. - if you are 15 days into the quarter you'll be 15 days into the resulting one. 		// bonus: this allows adding fractional quarters 		return this.addMonths(value * 3); 	};   	/** 	 * Adds the specified number of years to this instance.  	 * @param {Number}   The number of years to add. The number can be positive or negative [Required] 	 * @return {Date}    this 	 */ 	$P.addYears = function (value) { 		if (!value) { return this; } 		return this.addMonths(value * 12); 	};   	/** 	 * Adds (or subtracts) to the value of the years, months, weeks, days, hours, minutes, seconds, milliseconds of the date instance using given configuration object. Positive and Negative values allowed. 	 * Example 	<pre><code> 	Date.today().add( { days: 1, months: 1 } ) 	  	new Date().add( { years: -1 } ) 	</code></pre>  	 * @param {Object}   Configuration object containing attributes (months, days, etc.) 	 * @return {Date}    this 	 */ 	$P.add = function (config) { 		if (typeof config === "number") { 			this._orient = config; 			return this; 		} 		 		var x = config;   		Iif (x.day) { 			// If we should be a different date than today (eg: for 'tomorrow -1d', etc). 			// Should only effect parsing, not direct usage (eg, Finish and FinishExact) 			if ((x.day - this.getDate()) !== 0) { 				this.setDate(x.day); 			} 		} 		if (x.milliseconds) { 			this.addMilliseconds(x.milliseconds); 		} 		if (x.seconds) { 			this.addSeconds(x.seconds); 		} 		if (x.minutes) { 			this.addMinutes(x.minutes); 		} 		if (x.hours) { 			this.addHours(x.hours); 		} 		if (x.weeks) { 			this.addWeeks(x.weeks); 		} 		if (x.months) { 			this.addMonths(x.months); 		} 		if (x.years) { 			this.addYears(x.years); 		} 		if (x.days) { 			this.addDays(x.days); 		} 		return this; 	}; 	 	/** 	 * Get the week number. Week one (1) is the week which contains the first Thursday of the year. Monday is considered the first day of the week. 	 * The .getWeek() function does NOT convert the date to UTC. The local datetime is used.  	 * Please use .getISOWeek() to get the week of the UTC converted date. 	 * @return {Number}  1 to 53 	 */ 	$P.getWeek = function (utc) { 		// Create a copy of this date object   		var self, target = new Date(this.valueOf()); 		if (utc) { 			target.addMinutes(target.getTimezoneOffset()); 			self = target.clone(); 		} else { 			self = this; 		} 		// ISO week date weeks start on monday   		// so correct the day number   		var dayNr = (self.getDay() + 6) % 7; 		// ISO 8601 states that week 1 is the week   		// with the first thursday of that year.   		// Set the target date to the thursday in the target week   		target.setDate(target.getDate() - dayNr + 3); 		// Store the millisecond value of the target date   		var firstThursday = target.valueOf(); 		// Set the target to the first thursday of the year   		// First set the target to january first   		target.setMonth(0, 1); 		// Not a thursday? Correct the date to the next thursday   		Eif (target.getDay() !== 4) { 			target.setMonth(0, 1 + ((4 - target.getDay()) + 7) % 7); 		} 		// The weeknumber is the number of weeks between the    		// first thursday of the year and the thursday in the target week   		return 1 + Math.ceil((firstThursday - target) / 604800000); // 604800000 = 7 * 24 * 3600 * 1000   	}; 	 	/** 	 * Get the ISO 8601 week number. Week one ("01") is the week which contains the first Thursday of the year. Monday is considered the first day of the week. 	 * The .getISOWeek() function does convert the date to it's UTC value. Please use .getWeek() to get the week of the local date. 	 * @return {String}  "01" to "53" 	 */ 	$P.getISOWeek = function () { 		return p(this.getWeek(true)); 	};   	/** 	 * Moves the date to Monday of the week set. Week one (1) is the week which contains the first Thursday of the year. 	 * @param {Number}   A Number (1 to 53) that represents the week of the year. 	 * @return {Date}    this 	 */ 	$P.setWeek = function (n) { 		if ((n - this.getWeek()) === 0) { 			Iif (this.getDay() !== 1) { 				return this.moveToDayOfWeek(1, (this.getDay() > 1 ? -1 : 1)); 			} else { 				return this; 			} 		} else { 			return this.moveToDayOfWeek(1, (this.getDay() > 1 ? -1 : 1)).addWeeks(n - this.getWeek()); 		} 	};   	$P.setQuarter = function (qtr) { 		var month = Math.abs(((qtr-1) * 3) + 1); 		return this.setMonth(month, 1); 	};   	$P.getQuarter = function () { 		return Date.getQuarter(this); 	};   	$P.getDaysLeftInQuarter = function () { 		return Date.getDaysLeftInQuarter(this); 	};   	/** 	 * Moves the date to the next n'th occurrence of the dayOfWeek starting from the beginning of the month. The number (-1) is a magic number and will return the last occurrence of the dayOfWeek in the month. 	 * @param {Number}   The dayOfWeek to move to 	 * @param {Number}   The n'th occurrence to move to. Use (-1) to return the last occurrence in the month 	 * @return {Date}    this 	 */ 	$P.moveToNthOccurrence = function (dayOfWeek, occurrence) { 		if (dayOfWeek === "Weekday") { 			if (occurrence > 0) { 				this.moveToFirstDayOfMonth(); 				Eif (this.is().weekday()) { 					occurrence -= 1; 				} 			} else if (occurrence < 0) { 				this.moveToLastDayOfMonth(); 				if (this.is().weekday()) { 					occurrence += 1; 				} 			} else { 				return this; 			} 			return this.addWeekdays(occurrence); 		} 		var shift = 0; 		if (occurrence > 0) { 			shift = occurrence - 1; 		} 		else Eif (occurrence === -1) { 			this.moveToLastDayOfMonth(); 			if (this.getDay() !== dayOfWeek) { 				this.moveToDayOfWeek(dayOfWeek, -1); 			} 			return this; 		} 		return this.moveToFirstDayOfMonth().addDays(-1).moveToDayOfWeek(dayOfWeek, +1).addWeeks(shift); 	};     	var moveToN = function (getFunc, addFunc, nVal) { 		return function (value, orient) { 			var diff = (value - this[getFunc]() + nVal * (orient \|\| +1)) % nVal; 			return this[addFunc]((diff === 0) ? diff += nVal * (orient \|\| +1) : diff); 		}; 	}; 	/** 	 * Move to the next or last dayOfWeek based on the orient value. 	 * @param {Number}   The dayOfWeek to move to 	 * @param {Number}   Forward (+1) or Back (-1). Defaults to +1. [Optional] 	 * @return {Date}    this 	 */ 	$P.moveToDayOfWeek = moveToN("getDay", "addDays", 7); 	/** 	 * Move to the next or last month based on the orient value. 	 * @param {Number}   The month to move to. 0 = January, 11 = December 	 * @param {Number}   Forward (+1) or Back (-1). Defaults to +1. [Optional] 	 * @return {Date}    this 	 */ 	$P.moveToMonth = moveToN("getMonth", "addMonths", 12); 	/** 	 * Get the Ordinate of the current day ("th", "st", "rd"). 	 * @return {String}  	 */ 	$P.getOrdinate = function () { 		var num = this.getDate(); 		return ord(num); 	}; 	/** 	 * Get the Ordinal day (numeric day number) of the year, adjusted for leap year. 	 * @return {Number} 1 through 365 (366 in leap years) 	 */ 	$P.getOrdinalNumber = function () { 		return Math.ceil((this.clone().clearTime() - new Date(this.getFullYear(), 0, 1)) / 86400000) + 1; 	};   	/** 	 * Get the time zone abbreviation of the current date. 	 * @return {String} The abbreviated time zone name (e.g. "EST") 	 */ 	$P.getTimezone = function () { 		return $D.getTimezoneAbbreviation(this.getUTCOffset(), this.isDaylightSavingTime()); 	};   	$P.setTimezoneOffset = function (offset) { 		var here = this.getTimezoneOffset(), there = Number(offset) * -6 / 10; 		return (there \|\| there === 0) ? this.addMinutes(there - here) : this; 	};   	$P.setTimezone = function (offset) { 		return this.setTimezoneOffset($D.getTimezoneOffset(offset)); 	};   	/** 	 * Indicates whether Daylight Saving Time is observed in the current time zone. 	 * @return {Boolean} true\|false 	 */ 	$P.hasDaylightSavingTime = function () { 		return (Date.today().set({month: 0, day: 1}).getTimezoneOffset() !== Date.today().set({month: 6, day: 1}).getTimezoneOffset()); 	}; 	 	/** 	 * Indicates whether this Date instance is within the Daylight Saving Time range for the current time zone. 	 * @return {Boolean} true\|false 	 */ 	$P.isDaylightSavingTime = function () { 		return Date.today().set({month: 0, day: 1}).getTimezoneOffset() !== this.getTimezoneOffset(); 	};   	/** 	 * Get the offset from UTC of the current date. 	 * @return {String} The 4-character offset string prefixed with + or - (e.g. "-0500") 	 */ 	$P.getUTCOffset = function (offset) { 		var n = (offset \|\| this.getTimezoneOffset()) * -10 / 6, r; 		if (n < 0) { 			r = (n - 10000).toString(); 			return r.charAt(0) + r.substr(2); 		} else { 			r = (n + 10000).toString(); 			return "+" + r.substr(1); 		} 	};   	/** 	 * Returns the number of milliseconds between this date and date. 	 * @param {Date} Defaults to now 	 * @return {Number} The diff in milliseconds 	 */ 	$P.getElapsed = function (date) { 		return (date \|\| new Date()) - this; 	};   	/** 	 * Set the value of year, month, day, hour, minute, second, millisecond of date instance using given configuration object. 	 * Example 	<pre><code> 	Date.today().set( { day: 20, month: 1 } )   	new Date().set( { millisecond: 0 } ) 	</code></pre> 	 *  	 * @param {Object}   Configuration object containing attributes (month, day, etc.) 	 * @return {Date}    this 	 */ 	$P.set = function (config) { 		config = validateConfigObject.call(this, config); 		var key; 		for (key in config) { 			Eif (hasOwnProperty.call(config, key)) { 				var name = key.charAt(0).toUpperCase() + key.slice(1); 				var addFunc, getFunc; 				if (key !== "week" && key !== "month" && key !== "timezone" && key !== "timezoneOffset") { 					name += "s"; 				} 				addFunc = "add" + name; 				getFunc = "get" + name; 				if (key === "month") { 					addFunc = addFunc + "s"; 				} else if (key === "year"){ 					getFunc = "getFullYear"; 				} 				if (key !== "day" && key !== "timezone" && key !== "timezoneOffset"  && key !== "week" &&  key !== "hour") { 						this[addFunc](config[key] - this[getFunc]()); 				} else if ( key === "timezone"\|\| key === "timezoneOffset" \|\| key === "week" \|\| key === "hour") { 					this["set"+name](config[key]); 				} 			} 		} 		// day has to go last because you can't validate the day without first knowing the month 		if (config.day) { 			this.addDays(config.day - this.getDate()); 		} 		 		return this; 	};   	/** 	 * Moves the date to the first day of the month. 	 * @return {Date}    this 	 */ 	$P.moveToFirstDayOfMonth = function () { 		return this.set({ day: 1 }); 	};   	/** 	 * Moves the date to the last day of the month. 	 * @return {Date}    this 	 */ 	$P.moveToLastDayOfMonth = function () { 		return this.set({ day: $D.getDaysInMonth(this.getFullYear(), this.getMonth())}); 	};     	/** 	 * Converts the value of the current Date object to its equivalent string representation. 	 * Format Specifiers 	 * CUSTOM DATE AND TIME FORMAT STRINGS 	 * Format  Description                                                                  Example 	 * ------  ---------------------------------------------------------------------------  ----------------------- 	 * s      The seconds of the minute between 0-59.                                      "0" to "59" 	 * ss     The seconds of the minute with leading zero if required.                     "00" to "59" 	 *  	 * m      The minute of the hour between 0-59.                                         "0"  or "59" 	 * mm     The minute of the hour with leading zero if required.                        "00" or "59" 	 *  	 * h      The hour of the day between 1-12.                                            "1"  to "12" 	 * hh     The hour of the day with leading zero if required.                           "01" to "12" 	 *  	 * H      The hour of the day between 0-23.                                            "0"  to "23" 	 * HH     The hour of the day with leading zero if required.                           "00" to "23" 	 *  	 * d      The day of the month between 1 and 31.                                       "1"  to "31" 	 * dd     The day of the month with leading zero if required.                          "01" to "31" 	 * ddd    Abbreviated day name. Date.CultureInfo.abbreviatedDayNames.                                "Mon" to "Sun"  	 * dddd   The full day name. Date.CultureInfo.dayNames.                                              "Monday" to "Sunday" 	 *  	 * M      The month of the year between 1-12.                                          "1" to "12" 	 * MM     The month of the year with leading zero if required.                         "01" to "12" 	 * MMM    Abbreviated month name. Date.CultureInfo.abbreviatedMonthNames.                            "Jan" to "Dec" 	 * MMMM   The full month name. Date.CultureInfo.monthNames.                                          "January" to "December" 	 * 	 * yy     The year as a two-digit number.                                              "99" or "08" 	 * yyyy   The full four digit year.                                                    "1999" or "2008" 	 *  	 * t      Displays the first character of the A.M./P.M. designator.                    "A" or "P" 	 *		Date.CultureInfo.amDesignator or Date.CultureInfo.pmDesignator 	 * tt     Displays the A.M./P.M. designator.                                           "AM" or "PM" 	 *		Date.CultureInfo.amDesignator or Date.CultureInfo.pmDesignator 	 *  	 * S      The ordinal suffix ("st, "nd", "rd" or "th") of the current day.            "st, "nd", "rd" or "th" 	 * 	 * STANDARD DATE AND TIME FORMAT STRINGS 	 * Format  Description                                                                  Example 	 *------  ---------------------------------------------------------------------------  ----------------------- 	 * d      The CultureInfo shortDate Format Pattern                                     "M/d/yyyy" 	 * D      The CultureInfo longDate Format Pattern                                      "dddd, MMMM dd, yyyy" 	 * F      The CultureInfo fullDateTime Format Pattern                                  "dddd, MMMM dd, yyyy h:mm:ss tt" 	 * m      The CultureInfo monthDay Format Pattern                                      "MMMM dd" 	 * r      The CultureInfo rfc1123 Format Pattern                                       "ddd, dd MMM yyyy HH:mm:ss GMT" 	 * s      The CultureInfo sortableDateTime Format Pattern                              "yyyy-MM-ddTHH:mm:ss" 	 * t      The CultureInfo shortTime Format Pattern                                     "h:mm tt" 	 * T      The CultureInfo longTime Format Pattern                                      "h:mm:ss tt" 	 * u      The CultureInfo universalSortableDateTime Format Pattern                     "yyyy-MM-dd HH:mm:ssZ" 	 * y      The CultureInfo yearMonth Format Pattern                                     "MMMM, yyyy" 	 * 	 * @param {String}   A format string consisting of one or more format spcifiers [Optional]. 	 * @return {String}  A string representation of the current Date object. 	 */ 	 	var ord = function (n) { 		switch (n * 1) { 		case 1: 		case 21: 		case 31: 			return "st"; 		case 2: 		case 22: 			return "nd"; 		case 3: 		case 23: 			return "rd"; 		default: 			return "th"; 		} 	}; 	var parseStandardFormats = function (format) { 		var y, c = Date.CultureInfo.formatPatterns; 		switch (format) { 			case "d": 				return this.toString(c.shortDate); 			case "D": 				return this.toString(c.longDate); 			case "F": 				return this.toString(c.fullDateTime); 			case "m": 				return this.toString(c.monthDay); 			case "r": 			case "R": 				y = this.clone().addMinutes(this.getTimezoneOffset()); 				return y.toString(c.rfc1123) + " GMT"; 			case "s": 				return this.toString(c.sortableDateTime); 			case "t": 				return this.toString(c.shortTime); 			case "T": 				return this.toString(c.longTime); 			case "u": 				y = this.clone().addMinutes(this.getTimezoneOffset()); 				return y.toString(c.universalSortableDateTime); 			case "y": 				return this.toString(c.yearMonth); 			default: 				return false; 		} 	}; 	var parseFormatStringsClosure = function (context) { 		return function (m) { 			if (m.charAt(0) === "\\") { 				return m.replace("\\", ""); 			} 			switch (m) { 				case "hh": 					return p(context.getHours() < 13 ? (context.getHours() === 0 ? 12 : context.getHours()) : (context.getHours() - 12)); 				case "h": 					return context.getHours() < 13 ? (context.getHours() === 0 ? 12 : context.getHours()) : (context.getHours() - 12); 				case "HH": 					return p(context.getHours()); 				case "H": 					return context.getHours(); 				case "mm": 					return p(context.getMinutes()); 				case "m": 					return context.getMinutes(); 				case "ss": 					return p(context.getSeconds()); 				case "s": 					return context.getSeconds(); 				case "yyyy": 					return p(context.getFullYear(), 4); 				case "yy": 					return p(context.getFullYear()); 				case "y": 					return context.getFullYear(); 				case "E": 				case "dddd": 					return Date.CultureInfo.dayNames[context.getDay()]; 				case "ddd": 					return Date.CultureInfo.abbreviatedDayNames[context.getDay()]; 				case "dd": 					return p(context.getDate()); 				case "d": 					return context.getDate(); 				case "MMMM": 					return Date.CultureInfo.monthNames[context.getMonth()]; 				case "MMM": 					return Date.CultureInfo.abbreviatedMonthNames[context.getMonth()]; 				case "MM": 					return p((context.getMonth() + 1)); 				case "M": 					return context.getMonth() + 1; 				case "t": 					return context.getHours() < 12 ? Date.CultureInfo.amDesignator.substring(0, 1) : Date.CultureInfo.pmDesignator.substring(0, 1); 				case "tt": 					return context.getHours() < 12 ? Date.CultureInfo.amDesignator : Date.CultureInfo.pmDesignator; 				case "S": 					return ord(context.getDate()); 				case "W": 					return context.getWeek(); 				case "WW": 					return context.getISOWeek(); 				case "Q": 					return "Q" + context.getQuarter(); 				case "q": 					return String(context.getQuarter()); 				case "z": 					return context.getTimezone(); 				case "Z": 				case "X": 					return Date.getTimezoneOffset(context.getTimezone()); 				case "ZZ": // Timezone offset in seconds 					return context.getTimezoneOffset() * -60; 				case "u": 					return context.getDay(); 				case "L": 					return ($D.isLeapYear(context.getFullYear())) ? 1 : 0; 				case "B": 					// Swatch Internet Time (.beats) 					return "@"+((context.getUTCSeconds() + (context.getUTCMinutes()*60) + ((context.getUTCHours()+1)*3600))/86.4); 				default: 					return m; 			} 		}; 	}; 	$P.toString = function (format, ignoreStandards) { 		 		// Standard Date and Time Format Strings. Formats pulled from CultureInfo file and 		// may vary by culture.  		if (!ignoreStandards && format && format.length === 1) { 			output = parseStandardFormats.call(this, format); 			if (output) { 				return output; 			} 		} 		var parseFormatStrings = parseFormatStringsClosure(this); 		return format ? format.replace(/((\\)?(dd?d?d?\|MM?M?M?\|yy?y?y?\|hh?\|HH?\|mm?\|ss?\|tt?\|S\|q\|Q\|WW?W?W?)(?![^\[]*\]))/g, parseFormatStrings).replace(/\[\|\]/g, "") : this._toString(); 	};   }()); ``` |
```

Generated by istanbul at Sun Nov 16 2014 12:08:33 GMT-0800 (PST)
